# Acute family marital psychosis

**DOI:** 10.1192/j.eurpsy.2021.865

**Published:** 2021-08-13

**Authors:** D. Falfel, W. Homri, F. Ghrissi, M. Stambouli, M. Ben Bechir, L. Mouelhi, N. Bram, I. Ben Romdhane, R. Labbane

**Affiliations:** Psychiatry C, Razi hospital, Manouba, Tunisia

**Keywords:** acute psychotic episode, marital psychosis, nuptial psychosis, family psychosis

## Abstract

**Introduction:**

Acute marital psychosis is an acute psychotic episode occurring in a particular context which is marriage. In fact, marriage represents an event with a very important emotional load especially in the Arab-Muslim culture. This event can, in some people, induce a relapse of certain psychiatric disorders, particularly psychotic ones. We propose in this work to report two clinical observations concerning two brothers who both presented, a few years apart, an acute nuptial psychosis with two different evolutions.

**Objectives:**

Studing the characteristics of nuptial psychotic episode in a one family and the different evolution of each one.

**Methods:**

Reporting two clinical cases of two brothers who represented both acute psychotic episode in a nuptial context with different evolution.

**Results:**

The two patients are brothers with common psychiatric background which is their mother treated for chronic psychotic disorder. They were hospitalized in our psychiatric service for acute psychotic episode occuring in a nuptial context with chronic evolution for the first one and a partial recovery for the second one.

**Conclusions:**

Marriage represents an event with a very important emotional load especially in the Arab-Muslim culture. In thiscontext, acute psychotic episode can occur with different evolution for patients predisposed. Studing socio-cultral circumstances related to psychotic episode can help mental health professional to improve the quality of health care service.

